# Efficacy, tolerability and safety of darbepoetin alfa injection for the treatment of anemia associated with chronic kidney disease (CKD) undergoing dialysis: a randomized, phase-III trial

**DOI:** 10.1186/s12882-019-1209-1

**Published:** 2019-03-13

**Authors:** Shubhadeep D. Sinha, Vamsi Krishna Bandi, Bala Reddy Bheemareddy, Pankaj Thakur, Sreenivasa Chary, Kalpana Mehta, Vikranth Reddy Pinnamareddy, Rajendra Pandey, Subhramanyam Sreepada, Santosh Durugkar

**Affiliations:** 1 Clinical Development and Medical Affairs, Hetero Group, Hetero Corporate, 7-2-A2, Industrial Estates, Sanath Nagar, Hyderabad, Andhra Pradesh India; 2 Department of Nephrology, B.L.Y Nair Hospital, A.L Nair Road, Mumbai, Maharashtra India; 3 Care Hospitals, Road No. 1, Banjara Hills, Hyderabad, Andhra Pradesh India; 4 Department of Nephrology, Institute of Post Graduate Medical Education and Research Kolkata, 244 A.J.C Bose Road, Kolkata, West Bengal India; 5 Sri Raghavendra Hospital, 1-7-100, Opp. Round Building, Kamala Nagar, ECIL Cross Road, ECIL, Hyderabad, Andhra Pradesh 500062 India; 6 Ashwini Hospital and Ramakanth Heart Care Center, Shivaji Nagar, Nanded, Maharashtra India

**Keywords:** Darbepoetin alfa, Erythropoietin, Anemia, Dialysis, End-stage renal disease

## Abstract

**Background:**

Darbepoetin alfa (DA-α) is a long-acting erythropoiesis-stimulating glycoprotein which has half-life three-fold longer than that of Erythropoietin alfa (EPO). The objective of this study was to compare the efficacy and safety of DA-α injection versus EPO for treating renal anemia amongst Indian patients with end-stage renal disease (ESRD) undergoing dialysis.

**Methods:**

Patients of either gender (aged 18–65 years) with ESRD undergoing dialysis who had hemoglobin (Hb) levels < 10 g/dL after receiving EPO were switched to DA-α (0.45 μg/kg) once weekly subcutaneously or EPO 50 IU/kg thrice weekly subcutaneously (centrally randomized 1:1) for 12–24 weeks (correction phase) followed by 12 weeks maintenance phase (for Hb levels ≥10 g/dL). The primary efficacy endpoint was mean change in Hb level from baseline to end of correction phase.

**Results:**

In the intention-to-treat population (*n* = 126), the between group difference in mean Hb change was − 0.01 g/dL (95% CI – 0.68 to − 0.66, *p* = 0.97). After adjusting for covariates, the difference was − 0.2878 g/dL (95% CI -0.936 to0.360). The lower limit of the two-sided 95% CI of primary endpoint was above the pre-specified non-inferiority margin of − 1.0 g/dL. Similar trend of non-inferiority was observed for per-protocol population. Safety profile of DA-α and EPO were observed to be similar.

**Conclusion:**

Our study results demonstrated that for patients with ESRD undergoing dialysis, administering DA-α at lower dose frequency, is equally effective and well tolerated as EPO for treating renal anemia.

**Trial registration:**

CTRI/2012/07/002835 [Registered on: 27/07/2012]; Trial Registered Prospectively.

**Electronic supplementary material:**

The online version of this article (10.1186/s12882-019-1209-1) contains supplementary material, which is available to authorized users.

## Background

Anemia is an inevitable complication of chronic kidney disease (CKD) and is caused predominantly by insufficient production of erythropoietin from the failing kidneys and circulating levels of erythropoietin [1–3]. In patients with CKD, untreated anemia has been associated with poor outcomes such as deterioration of cardiac function, decreased cognition, mental acuity, fatigue, and mortality [2–5].

Erythropoietin alfa (EPO) is a short acting erythropoiesis-stimulating agent (ESA) and has been a primary choice for treating anemia in patients with CKD for the past twenty years [6]. EPO is approved for the correction of anemia in patients with chronic renal failure, and its use in renal anemia eliminates the need for red cell transfusions, alleviates the symptoms of anemia, improves survival, reduces cardiovascular morbidity, and enhances the quality of life [6, 7]. However, its optimal route of administration and dosage are debatable due to its short half-life [8]. Intravenous (IV) administration of EPO should be limited to haemodialysis patients at a dosage of three times per week, as any dose reduction can lead to a major increase in the dose requirement [8]. In addition, high doses of epoetin alfa increase the risk of poor outcomes including cardiovascular events [6]. On the other hand, subcutaneous (sc) EPO therapy is efficacious during hemodialysis, peritoneal dialysis, and pre-dialysis in patients with CKD [8]. The frequent dosing regimen of EPO poses a constant strain on patients and health care staff. Thus, long-acting ESAs are favored over short-acting ESAs [9].

Darbepoetin alfa (DA-α manufactured by Hetero Biopharma) is the first long-acting ESA with extended dosing intervals and thus has an advantage over epoetins alfa and beta. DA-α has played an important role in the effective management of anemia and is preferred over epoetins/biosimilar epoetins for patients requiring less-frequent administration of ESAs [6]. DA-α owing to its longer half-life, maintains target hemoglobin levels (10-12g/dL) with low dosing frequency (once weekly or biweekly), benefiting patients and health care staff equally [6–9]. The dose requirement of EPO by the sc route was 22% lesser than that by the IV route; however, DA-α has similar dose requirements by both the sc and IV routes, which simplifies management of anemia [6].

Clinical studies have shown that DA-α administered once every 2 weeks or every month improves convenience and saves costs with no compromise in its efficacy while maintaining the target hemoglobin (Hb) range in patients [6]. Therefore, this study aimed to determine whether a biosimilar DA-α has similar efficacy and safety as that of EPO when given at a reduced dose frequency for the treatment of renal anemia in Indian patients with end-stage renal disease (ESRD) undergoing dialysis.

## Methods

The manuscript has been prepared as per CONSORT extension for non-inferiority and equivalence trial.

### Study design and patient selection

This was a prospective phase III, randomized, open label, two-arm, parallel group, multi-center, active-controlled, noninferiority clinical study performed at 14 nephrology centers in India during Sept 2012–May 2014. Permuted block randomization schedule with block of size 4 and ratio of 1:1 in the two groups was generated using SAS® software version 9.1. The study was not blinded because of highly varying dosing schedules in the treatment groups. In this study, clinically stable patients (M/F) aged 18–65 years who were on hemodialysis or peritoneal dialysis for at least 4 weeks and had baseline Hb levels < 12 g/dL, were enrolled. Study included patients who were on EPO (not within 1 week before screening) or those who were EPO - naive, and had adequate serum ferritin (≥ 200 ng/mL) and transferrin saturation (≥ 20%) levels. Major exclusion criteria were congestive heart failure, history of uncontrolled hypertension (not amenable two standard drugs over 2 weeks of screening period), severe hyperparathyroidism, pregnant women or lactating mothers, diabetes with HbA1C ≥ 10%, systemic hematological diseases, liver disease, reported hypersensitive to any active study drug substances, or infections. During the study Vitamin B12 and red cell folate concentrations were tested at screening, week 12, end of correction phase and end of maintenance phase. Iron tests i.e. Serum Ferritin, Iron, TIBC, and TSAT were measured at screening, weeks 4, 8, 12, 16, 20, 24 and end of maintenance. Inflammatory marker (CRP) is measured at screening.

The study treatment comprised correction phase (12 to 24 weeks) and maintenance phase (24 to 36 weeks). During 12-24 weeks of correction phase, at baseline, the patients having Hb levels < 10 g/dL with EPO, were switched to either DA-α once weekly (0.45 μg/kg subcutaneous injection manufactured by Hetero Biopharma Limited, India) or EPO thrice weekly (50 IU/kg, Eprex® manufactured by Cilag AG, Switzerland) in allocation ratio of 1:1. The patients with treatment failure, i.e those who had Hb level < 10 g/dL at the end of correction phase (EOC), were discontinued from the study. Following EOC phase, the patients with Hb levels ≥10 g/dL were switched to DA-α for 12 weeks of maintenance phase. If the dialysis patients had Hb level ≥ 10 g/dL at baseline, they directly entered the maintenance phase and were randomized (1:1) to receive DA-α (0.45 μg/kg) once weekly or EPO (50 IU/kg) thrice weekly. In both of the treatment arms, appropriate dose adjustments were made to achieve and maintain patients’ Hb level within the target range, i.e ≥ 1 g/dL increase from baseline Hb, and within the range of 10–12 g/dL during the 36-week study period. Dosage was increased by 25% if a patient’s Hb remained < 10 g/dL even after achieving the target range during the correction phase. In the maintenance phase, if the patient’s Hb levels went above the target range (≥ 11.5 g/dL) for two consecutive weekly assessments, the dosage was decreased by 25%. The dosage was increased by 25%, if after achieving the target range (10–12 g/dL), patient’s Hb level was below 10 g/dL. There was no specific rescue therapy defined in the study protocol. However, Iron supplements (oral/IV) were allowed as concomitant medication to prevent apparent iron deficiency. For patients with serum ferritin values < 100 μg/L or ≥ 100 μg/L, the IV iron dosing regimen was determined per the individual center’s treatment protocol.

### Efficacy and safety assessments

Hemoglobin (Hb) levels were assessed throughout the correction and maintenance phases. Primary efficacy endpoint included the mean change in Hb level from baseline at the first evaluation visit (EOC) in patients treated with EPO versus DA-α. Secondary efficacy endpoints were Hb variability during correction phase, mean change in Hb levels from baseline to week-4 and week-36 (EOM), proportion of patients achieving the Hb target (defined as Hb increase ≥ 1 g/dL from baseline) at EOC, mean DA-α dose, proportion of patients who could maintain the target Hb of 10–12 g/dL at EOM, and time to initial achievement of Hb target. Safety assessment included serum chemistry, complete blood count, and urinalysis measured at baseline and after treatment. Adverse events (AEs) were monitored at each study visit. Safety endpoints were the incidence of treatmentemergent adverse events (TEAEs) and immunogenicity as assessed by anti-drug antibody titers using ELISA methods. Samples for immunogenicity to DA-α/EPO were withdrawn within 1 h of dosing before initial dosing, i.e Day 1 of week 1 and on day 1 of weeks 5, 13, 25 and EOM. Since the screening time and evaluation periods of the various patient subgroups were different, the time points for immunogenicity varied accordingly.

### Statistical analyses

Assuming an estimated difference between the treatments of − 0.5 g/dL in mean change in Hb levels with a noninferiority margin at − 1.0 g/dL, to achieve 80% power for the non-inferiority test, a total of 100 patients are required to be allocated in the ratio of 1:1 to either DA-α (*n* = 50) or EPO (*n* = 50). A final sample size of 126 patients (63 patients in each treatment arm) was derived assuming dropout rate at 20%. A designated statistician (Sristek, India) generated the allocation sequence and assigned participants to their groups, and investigators at 14 nephrology sites enrolled participants according to this sequence. Efficacy analysis included both the intent to treat (ITT) and per protocol (PP) population. ITT population comprised randomized patients who received at least one dose of the study drug, with baseline and at least one efficacy assessment available during the evaluation period. PP set included patients who completed all the study visits as defined in the protocol without major protocol deviations. Safety population comprised all randomized patients who received at least one dose of the study drug. The between group difference in the mean Hb change from baseline to first evaluation visit was analyzed using analysis of covariance (ANCOVA). Treatment was considered as main effect and baseline Hb levels as the covariate in the model. The two-sided 95% CI for difference in mean Hb for treatments was calculated to assess the non-inferiority, and if it was above the non-inferiority margin of − 1.0 g/dL, the non-inferiority was accepted. Two sample t-tests at 5% level of significance were performed to compare the difference of change in Hb level from baseline to week-4 and week-36 EOM. For all tests, *p*-value of ≤ 0.05 was considered as statistically significant. Logistic regression was carried out to compare the proportion of patients achieving the Hb target (≥ 1 g/dL from baseline and Hb concentration of 10–12 g/dL) at the end of first evaluation visit and EOM. The time to the initial achievement of Hb target at the EOC was estimated using Kaplan-Meier. SAS® Version 9.4 (SAS Institute Inc., USA) was used to perform all statistical analyses.

## Results

### Patient disposition and baseline characteristics

**Fig. 1:**
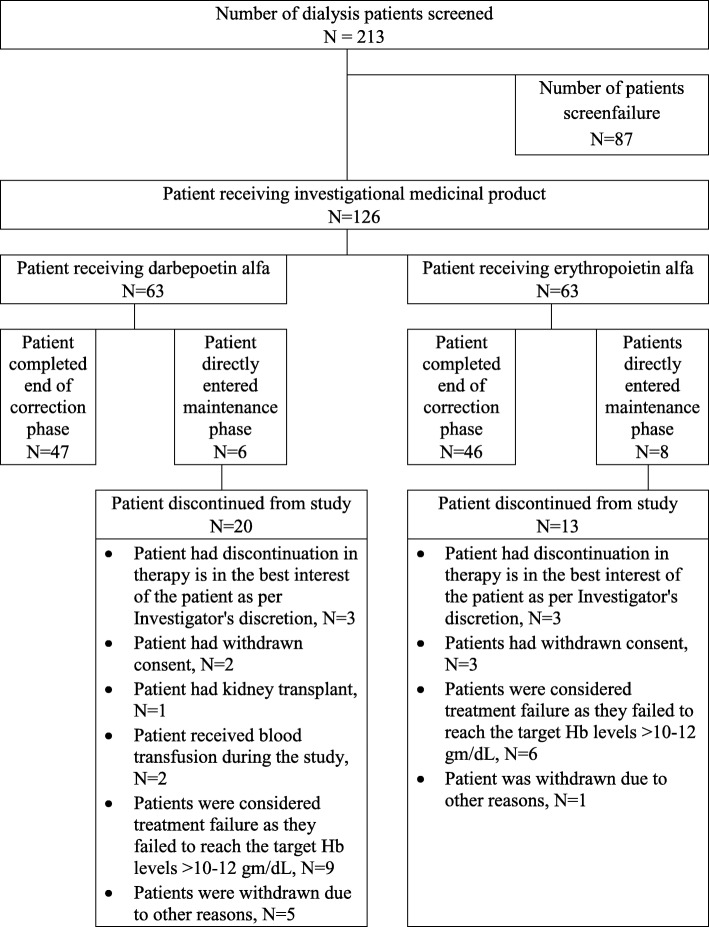
Patients Flow

**Fig. 2:**
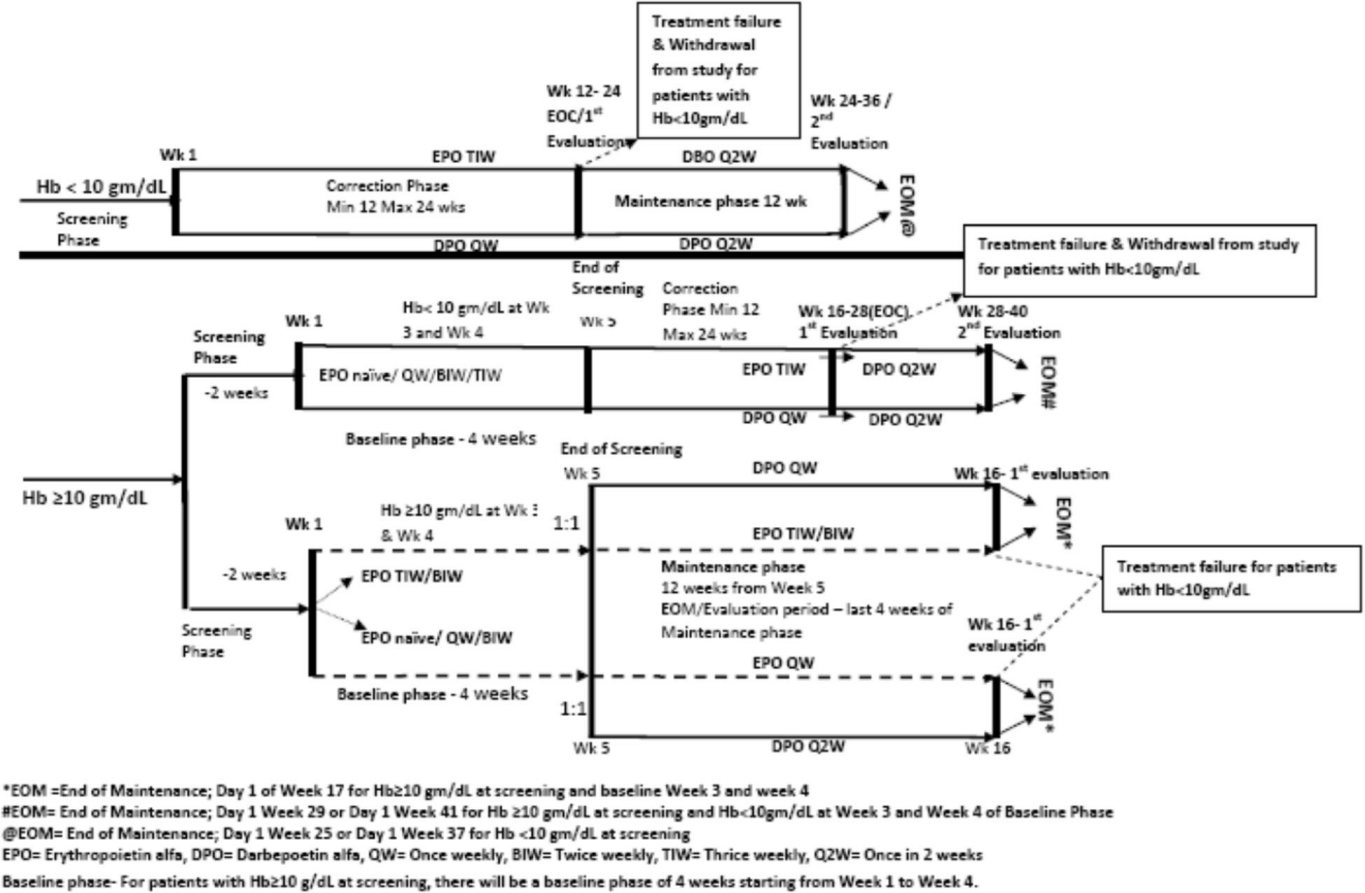
Schematic diagram of study design

A total of 213 patients who underwent dialysis were screened for anaemia (Fig. 1). Of these, a total of 126 patients (63 patients in each group) who met the eligibility criteria were enrolled and randomized (ITT population). The PP population consisted of 107 patients, of which 93 patients were in correction phase due to Hb level < 10 g/dL at screening or baseline, 14 patients directly entered into the maintenance phase due to Hb level > 10 g/dL at screening and baseline who were on EPO (thrice weekly/biweekly) during screening & baseline phase. The demographic characteristics of the safety population are given in Table 1. Patient’s flow in the study as well as schematic diagram of the study elaborated in Figs. 1 and 2, respectively. Majority of patients were male in both the treatment groups, where the mean (± SD) age of enrolled patients was 46.8 (± 12.32). The mean (± SD) dose of DA-α was 26.12 (± 0.98) μg at week 1 and 27.73 (± 1.62) μg at week 12. The mean (± SD) dose of EPO at week 1 and 12 was 3038.3 (± 115.42) IU and 2998.7 (± 221.23) IU, respectively.

**Table 1:** Demographic characteristics (ITT population)

Variable	Darbepoetin alfa (*N* = 63)	Erythropoietin alfa (*N* = 63)	Overall (*N* = 126)
Age (years)			
Mean (SD)	44.8 (11.81)	48.8 (12.60)	46.8 (12.32)
Median (range)	47.0 (21, 65)	51.0 (21, 65)	49.0 (21, 65)
Height (cm)			
Mean (SD)	158.6 (11.31)	159.5 (22.27)	159.1 (17.60)
Median (range)	160.0 (127, 178)	163.0 (5.5, 182)	160.0 (5.5, 182)
Weight (kg)			
Mean (SD)	58.93 (15.30)	58.74 (11.92)	58.84 (13.66)
Median (range)	56.50 (34, 95)	60.00 (38.5, 88)	57.50 (34, 95)
Gender, n (%)			
Male	39 (61.90)	46 (73.02)	85 (67.46)
Female	24 (38.10)	17 (26.98)	41 (32.54)

*n* number of subject at each visit; *N* total number of subjects, *ITT* Intent to treat

### Efficacy assessment

In ITT population, Hb levels were increased gradually from baseline to the end of first evaluation period, with the mean change in Hb levels of 1.84 and 1.85 in DA-α and EPO group, respectively (within group comparison, *p* < 0.001 for each). Change from baseline to the end of first evaluation period in Hb levels was equal in PP population for both treatment groups. The mean Hb change from baseline to the first evaluation period was equal in both, DA-α (1.84; 95% CI 1.36 to 2.32) and EPO (1.85; 95% CI 1.37 to 2.33) groups (Table 2). The difference in the mean change in Hb levels amongst the two groups was − 0.01 g/dL (95% CI − 0.68 to − 0.66, *p* = 0.97). The difference was not statistically significant even with the low frequency of DA-α administration (Table 2).

**Table 2:** Mean Hb levels (g/dL) and mean change in hemoglobin from Baseline to EOC – Dialysis, ITT Population (*N* = 126)

Statistics	ITT Population (*N* = 126)		PP Population (*N* = 93)	
	Darbepoetin alfa (*n* = 63)	Erythropoietin alfa (*n* = 63)	Darbepoetin alfa (*n* = 47)	Erythropoietin alfa (*n* = 46)
Baseline				
n	56	53	47	46
Mean (SD)	8.39 (0.90)	8.80 (0.89)	8.39 (0.85)	8.72 (0.91)
End of first evaluation visit				
n	55	51	47	46
Mean (SD)	10.20 (1.74)	10.61 (1.55)	10.33 (1.42)	10.90 (0.95)
Within group comparison				
*p*-value#	<.0001	<.0001	<.0001	<.0001
Mean change	1.84	1.85	1.94	2.18
95% CI	[1.36–2.32]	[1.37–2.33]	[1.48–2.40]	[1.84–2.53]
Between group comparison				
Mean change	−0.01		−0.24	
95% CI	[−0.68–0.66]		[− 0.81–0.32]	
*p*-value**	0.9703		0.3985	

*N* number of subject at each visit, *N* total number of subjects, *ITT* Intent to treat, *PP* Per protocol

# *p*-values were obtained using Paired t Test for mean (two tailed, α 0.05)

** *p*-values were obtained using Unpaired t Test for mean change (two tailed, α = 0.05)

Note: Patients taken where Hb < 10 at Screening

The difference in the mean Hb change amongst the two groups was − 0.2878 g/dL (95% CI − 0.936 to − 0.360) when adjusted for covariates (using ANCOVA) (Table 3). The lower limit of the two-sided 95% CI of primary endpoint was above the pre-specified non-inferiority margin of − 1.0 g/dL, irrespective of being adjusted (− 0.936) or unadjusted (− 0.68) for covariates, establishing that DA-α was equally effective as EPO, in maintaining mean Hb despite the reduced dosing frequency in patients undergoing dialysis. The results of PP population analysis set further confirmed the robustness of ITT analysis (Tables 2 and 3). In PP population, the lower limit of the 95% CI was − 0.994 (adjusted) and − 0.81 (unadjusted), which was above the pre-specified non-inferiority margin of − 1.0 g/dL.

**Table 3:** Adjusted mean change in hemoglobin levels (g/dL) from baseline to first evaluation period (EOC)

Coefficient	Estimate	Standard Error	95% CI of Mean	*p*-value*
ITT Population (*N* = 126)				
Baseline Value	− 0.6799	0.18076	[−1.038,−0.321]	0.0003
Darbepoetin alfa	−0.2878	0.32675	[−0.936,−0.360]	0.3805
Erythropoietin alfa	0.0000	–	–	–
PP Population (*N* = 93)				
Baseline Value	−0.7725	0.142555	[−1.056,−0.489]	<.0001
Darbepoetin alfa	−0.4917	0.253015	[−0.994,−0.011]	0.0551
Erythropoietin alfa	0.0000	–	–	–

Model: Change in Hb levels = Treatment Group+ Site + Baseline value

*p-value was calculated using ANCOVA (two tailed, α = 0.05)

#### Secondary analyses

Improvement in Hb levels was observed as early as week 4 in both treatment groups which showed similar mean Hb change in the DA-α (0.30 g/dL, 95% CI − 0.01 to − 0.61, *p* = 0.0566) and EPO (0.74 g/dL, 95% CI 0.29 to 1.19, *p* = 0.0019) groups (Table 4). The difference in mean Hb change between the two groups was − 0.44 g/dL (95% CI − 0.97 to − 0.09, *p* = 0.105), which was not statistically significant irrespective of the reduced frequency of DA-α administration. Increase in Hb levels from baseline was also similar in PP population even at week 4 (mean Hb change: − 0.62, 95% CI − 1.14 to − 0.10, *p* = 0.0209). The between group comparison at the end of second evaluation visit showed that the difference in mean Hb change was similar in both ITT (*p* = 0.94) and PP population (*p* = 0.88).

**Table 4:** Mean change in hemoglobin levels (g/dL) from baseline to week-4

Statistics	ITT Population (*N* = 126)		PP Population (*N* = 93)	
	Darbepoetin alfa (*n* = 63)	Erythropoietin alfa (*n* = 63)	Darbepoetin alfa (*n* = 47)	Erythropoietin alfa (*n* = 46)
Baseline				
n	56	53	47	46
Mean (SD)	8.39 (0.90)	8.80 (0.89)	8.39 (0.85)	8.72 (0.91)
Week-4				
n	55	50	47	45
Mean (SD)	8.66 (1.24)	9.50 (1.81)	8.68 (1.13)	9.62 (1.71)
Within group comparison				
*p*-value*	0.0566	0.0019	0.0473	0.0002
Mean change	0.30	0.74	0.29	0.91
95% CI	[−0.01–0.61]	[0.29–1.19]	[0.00–0.57]	[0.45–1.36]
Between group comparison				
Mean change	−0.44		−0.62	
95% CI	[−0.97–0.09]		[−1.14−0.10]	
p-value**	0.1057		0.0209	

*n* number of subject at each visit; *N* total number of subjects, *ITT* Intent to treat, *PP* Per protocol

* *p*-value were obtained using Paired t Test for mean (two tailed, a = 0.05)

**p-value were obtained using Unpaired t Test for mean change (two tailed, a = 0.05)

Note: Patients taken where Hb < 10 at Screening

As summarized in Table 5, in ITT population, the similar proportions of patients were observed to achieve target Hb level at the end of the first evaluation visit (DA-α vs. EPO: 52.38% vs. 49.2%; OR [95% CI] = 0.96 [0.46 to 1.99], *p* = 0.90). Similar results was observed in PP population (DA-α vs. EPO: 68.08% vs. 69.56%; OR [95% CI] = 0.94 [0.39 to 2.30], *p* = 0.89). In ITT and PP population, the KM estimated median time to achieve the target Hb level was 9 weeks and 7 weeks after DA-α and EPO treatment, respectively. In ITT population, the proportion of patients who maintained their Hb levels within the target Hb range (10–12 g/dL) till the EOM was similar in both the treatment groups (DA-α vs. EPO: 38.10% vs. 57.14% respectively; OR [95% 314 CI] = 0.57 [0.26 to1.25], *p* = 0.16). Similar result was observed in PP population for both treatment groups (DA-α vs. EPO: 34.09% vs. 57.50% respectively; OR [95% CI] = 0.46 [0.17 to 1.22], *p* = 0.11). This demonstrates that, compared to EPO, DA-α does not increase Hb variability despite reducing the dosing frequency. Furthermore, fewer dose adjustments were observed in DA-α treated patients.

**Table 5:** Time to initially attained target Hb level (10–12 g/dL) and proportion of patients attained target Hb level (10–12 g/dL) at EOC and EOM

Parameter	ITT Population (*N* = 126)		PP Population (*N* = 93)	
	Darbepoetin alfa (*n* = 63)	Erythropoietin alfa (n = 63)	Darbepoetin alfa (*n* = 47)	Erythropoietin alfa (*n* = 46)
Number of weeks to initially attain target Hb				
Median (95%CI)	9.00 (7.00–11.00)	7.00 (4.00–9.00)	9.00 (7.00–10.00)	7.00 (4.00–8.00)
No. of Patients initially attained target Hb level				
N (%)	44 (78.57)	43 (82.69)	40 (85.10)	41 (89.13)
Hazard Ratio (95%CI)	0.807 (0.53–1.23)		0.778 (0.50–1.21)	
*P* Value	0.3212		0.2608	
No. of patients attained target Hb level at EOC				
N (%)	33 (52.38)	31 (49.2)	32 (68.08)	32 (69.56)
Odd ratios (95%CI)	0.9559 (0.46–1.99)		0.9410 (0.39–2.30)	
*P* value	0.9038		0.8938	
No. of patients maintained target Hb level at EOM				
(%)	24 (38.10)	36 (57.14)	15 (34.09)	23 (57.50)
Odd ratios (95%CI)	0.5748 (0.26–1.25)		0.4567 (0.17–1.22)	
*P* Value	0.1621		0.1180	

*EOC* End of correction, *EOM* End of maintenance, *ITT* Intent to treat, *PP* Per protocol, *Hb* Hemoglobin

#### Safety assessment

In the DA-α group, 25 (39.7%) patients and 32 (50.8%) patients in EPO group experienced at least one TEAE during the study period. Patients mostly reported TEAEs of mild-to-moderately severe in nature except one (1.58%) from the EPO group who experienced severe TEAEs. None from the DA-α group reported any TEAE related to the study drug; whereas, 4 patients from the EPO group reported 5 AE related to the study drug. In the EPO group, 4 patients reported 5 AEs that were related to the study drug. SAEs were reported by 9 patients (5 from EPO group and 4 from the DA-α group). The commonly reported events in both the treatment groups were pyrexia (DA-α vs. EPO: 9.5% vs. 7.9%), cough (9.5% vs. 15.9%), vomiting (4.8% vs. 6.3%), nasopharyngitis (4.8% vs. 6.3%), increased blood creatinine and urea (4.8% vs. 4.8% for each) and decreased glomerular filtration rate (4.8% vs. 4.8%). During the study, clinical laboratory evaluation of hematology, biochemistry and coagulation indicated no unexpected changes which could be attributed to the study drug. During the study, no changes in vital signs, mean blood pressure or heart rate were observed in either of the treatment groups.

Treatment emergent adverse events (TEAEs) were evaluated throughout the study. In DA-α group, the most commonly reported TEAEs by SOC were; respiratory, thoracic and mediastinal disorders (14.3%), general disorders and administration site conditions (12.7%), investigations (9.5%), and infections and infestations (7.9%). In EPO group, the most commonly reported TEAEs by SOC were; respiratory, thoracic and mediastinal disorders (22.2%), general disorders and administration site conditions (15.9%), vascular disorders (12.7%), gastrointestinal disorders (11.1%), infections and infestations (9.5%), and investigations (7.9%). There was no statistical difference between the two groups in terms of TEAEs.

Altogether, DA-α had a similar safety profile to that of EPO and no antibody formation was identified.

## Discussion

This randomized, active-controlled study aimed to compare the efficacy and safety of DA-α and EPO for the treatment of renal anemia among Indian patients with ESRD (end-stage renal disease) undergoing dialysis. This study achieved its primary efficacy, which demonstrates non-inferiority of DA-α over a most-widely used comparator, EPO. The results of this study demonstrated that, the efficacy of DA-α, when administered at reduced dose frequency, is similar to EPO for treating renal anaemia in patients undergoing dialysis. In this study, patients who were treated with DA-α could effectively maintain their Hb levels in the target therapeutic ranges and required with fewer dose adjustments than those treated with EPO. Therefore, the treatment using DA-α rules out the need for frequent monitoring and dose adjustments. In addition, both groups showed similar increases in Hb levels. Evaluating the iron availability for erythropoeisis is crucial in treating anaemia patients with CKD.Iron deficiency can interfere with the response to EPO and DA-α and affecting the efficacy. Thus, according to clinical practice guidelines and recommendations, iron supplements were given to all the patients in our study [4, 10]. To help maintain serum ferritin within recommended levels, most patients from both treatment arms received iron supplements. Additionally, serum ferritin levels were similar in both treatment groups.

In our study, DA-α (manufactured by Hetero Biopharma) was as safe as EPO. A majority of the reported AEs were due to the underlying disease and its treatment; in addition, only few AEs were associated with EPO use, and none were related to DA-α use. This study showed that safety profile of DA-α similar to those in other clinical trials conducted in pre dialysis stage of patients [11–13]. Also, it was observed that DA-α was well tolerated and had similar safety profile to EPO.

## Conclusion

The results of our study demonstrated that efficacy and safety of DA-α (manufactured by Hetero Biopharma), when administered at a reduced dose frequency, is similar to EPO for treating renal anemia in Indian patients with ESRD undergoing dialysis.

## Additional file

Additional file 1: **Table S1.** List of Institutional Ethics Committee. (DOCX 14 kb)

## Abbreviations

AEs: Adverse events
ANCOVA: Analysis of covariance
CI: Confidence interval
CKD: Chronic kidney disease
DA-α: Darbepoetin alfa
ELISA: Enzyme linked immunosorbent assay
EOC: End of correction
EOM: End of maintenance
EPO: Erythropoietin alfa
ESA: Erythropoiesis-stimulating agent
ESRD: End-stage renal disease
Hb: Hemoglobin
ITT: Intent to treat
IV: Intravenous
PP: Per protocol
sc: Subcutaneous
SD: Standard deviation
TEAEs: Treatment-emergent adverse events
