# Correction to: Efficacy, tolerability and safety of darbepoetin alfa injection for the treatment of anemia associated with chronic kidney disease (CKD) undergoing dialysis: a randomized, phase-III trial

**DOI:** 10.1186/s12882-019-1515-7

**Published:** 2019-11-19

**Authors:** Shubhadeep D. Sinha, Vamsi Krishna Bandi, Bala Reddy Bheemareddy, Pankaj Thakur, Sreenivasa Chary, Kalpana Mehta, Vikranth Reddy Pinnamareddy, Rajendra Pandey, Subhramanyam Sreepada, Santosh Durugkar

**Affiliations:** 1 Clinical Development and Medical Affairs, Hetero Group, Hetero Corporate, 7-2-A2, Industrial Estates, Sanath Nagar, Hyderabad, Andhra Pradesh India; 2 Department of Nephrology, B.L.Y Nair Hospital, A.L Nair Road, Mumbai, Maharashtra India; 3 Care Hospitals, Road No. 1, Banjara Hills, Hyderabad, Andhra Pradesh India; 4 Department of Nephrology, Institute of Post Graduate Medical Education and Research Kolkata, 244 A.J.C Bose Road, Kolkata, West Bengal India; 5 Sri Raghavendra Hospital, 1-7-100, Opp. Round Building, Kamala Nagar, ECIL Cross Road, ECIL, Hyderabad, Andhra Pradesh 500062 India; 6 Ashwini Hospital and Ramakanth Heart Care Center, Shivaji Nagar, Nanded, Maharashtra India

**Correction to: BMC Nephrol (2019) 20:90**

**https://doi.org/10.1186/s12882-019-1209-1**

Following publication of the original article [1], the authors reported errors in the presentation of Tables 2, 4 and 5. Additionally, the authors reported an error in the last paragraph of the ‘Safety assessment’ section and an error in the first paragraph of the ‘Discussion’ section. In this Correction the incorrect and correct version of Tables 2, 4 and 5 and the incorrect and correct version of the sentences in the ‘Safety assessment’ and ‘Discussion’ section are shown.

Originally Table 2 was published as:

**Table 2:** Mean Hb levels (g/dL) and mean change in hemoglobin from Baseline to EOC – Dialysis, ITT Population (*N* = 126)

Statistics	ITT Population (*N* = 126)		PP Population (*N* = 93)	
	Darbepoetin alfa (*n* = 63)	Erythropoietin alfa (*n* = 63)	Darbepoetin alfa (*n* = 47)	Erythropoietin alfa (*n* = 46)
Baseline				
n	56	53	47	46
Mean (SD)	8.39 (0.90)	8.80 (0.89)	8.39 (0.85)	8.72 (0.91)
End of first evaluation visit				
n	55	51	47	46
Mean (SD)	10.20 (1.74)	10.61 (1.55)	10.33 (1.42)	10.90 (0.95)
Within group comparison				
*p*-value#	<.0001	<.0001	<.0001	<.0001
Mean change	1.84	1.85	1.94	2.18
95% CI	[1.36–2.32]	[1.37–2.33]	[1.48–2.40]	[1.84–2.53]
Between group comparison				
Mean change	−0.01		−0.24	
95% CI	[−0.68–0.66]		[− 0.81–0.32]	
*p*-value**	0.9703		0.3985	

*N* number of subject at each visit, *N* total number of subjects, *ITT* Intent to treat, *PP* Per protocol

^#^ *p*-values were obtained using Paired t Test for mean (two tailed, α 0.05)

^**^ *p*-values were obtained using Unpaired t Test for mean change (two tailed, α = 0.05)

Note: Patients taken where Hb < 10 at Screening

The correct version of Table 2, with the corrected sections indicated in bold:

**Table 2:** Mean Hb levels (g/dL) and mean change in hemoglobin from Baseline to EOC – Dialysis, ITT Population (*N* = 126)

Statistics	ITT Population (*N* = 126)		PP Population (*N* = 93)	
	Darbepoetin alfa (*n* = 63)	Erythropoietin alfa (*n* = 63)	Darbepoetin alfa (*n* = 47)	Erythropoietin alfa (*n* = 46)
Baseline				
n	56	53	47	46
Mean (SD)	8.39 (0.90)	8.80 (0.89)	8.39 (0.85)	8.72 (0.91)
End of first evaluation visit				
n	55	51	47	46
Mean (SD)	10.20 (1.74)	10.61 (1.55)	10.33 (1.42)	10.90 (0.95)
Within group comparison				
*p*-value^#^	<.0001	<.0001	<.0001	<.0001
Mean change	1.84	1.85	1.94	2.18
95% CI	[1.36–2.32]	[1.37–2.33]	[1.48–2.40]	[1.84–2.53]
Between group comparison				
Mean change	−0.01		−0.24	
95% CI	[−0.68–0.66]		[− 0.81–0.32]	
*p*-value^**^	0.9703		0.3985	

***n*** number of subject at each visit, *N* total number of subjects, *ITT* Intent to treat, *PP* Per protocol, ***Hb*** **Hemoglobin**

^#^ *p*-values were obtained using Paired t Test for mean (two tailed, **α = 0.05**)

^**^ *p*-values were obtained using Unpaired t Test for mean change (two tailed, α = 0.05)

Note: Patients taken where Hb < 10 at Screening

Originally Table 4 was published as:

**Table 4:** Mean change in hemoglobin levels (g/dL) from baseline to week-4

Statistics	ITT Population (*N* = 126)		PP Population (*N* = 93)	
	Darbepoetin alfa (*n* = 63)	Erythropoietin alfa (*n* = 63)	Darbepoetin alfa (*n* = 47)	Erythropoietin alfa (*n* = 46)
Baseline				
n	56	53	47	46
Mean (SD)	8.39 (0.90)	8.80 (0.89)	8.39 (0.85)	8.72 (0.91)
Week-4				
n	55	50	47	45
Mean (SD)	8.66 (1.24)	9.50 (1.81)	8.68 (1.13)	9.62 (1.71)
Within group comparison				
*p*-value^*^	0.0566	0.0019	0.0473	0.0002
Mean change	0.30	0.74	0.29	0.91
95% CI	[− 0.01–0.61]	[0.29–1.19]	[0.00–0.57]	[0.45–1.36]
Between group comparison				
Mean change	−0.44		−0.62	
95% CI	[−0.97–0.09]		[−1.14–0.10]	
*p*-value^**^	0.1057		0.0209	

*n* number of subject at each visit; *N* total number of subjects, *ITT* Intent to treat, *PP* Per protocol

^*^ *p*-value were obtained using Paired t Test for mean (two tailed, a = 0.05)

^**^p-value were obtained using Unpaired t Test for mean change (two tailed, a = 0.05)

Note: Patients taken where Hb < 10 at Screening

The correct version of Table 4, with the corrected sections indicated in bold:

**Table 4:** Mean change in hemoglobin levels (g/dL) from baseline to week-4

Statistics	ITT Population (*N* = 126)		PP Population (*N* = 93)	
	Darbepoetin alfa (*n* = 63)	Erythropoietin alfa (*n* = 63)	Darbepoetin alfa (*n* = 47)	Erythropoietin alfa (*n* = 46)
Baseline				
n	56	53	47	46
Mean (SD)	8.39 (0.90)	8.80 (0.89)	8.39 (0.85)	8.72 (0.91)
Week-4				
n	55	50	47	45
Mean (SD)	8.66 (1.24)	9.50 (1.81)	8.68 (1.13)	9.62 (1.71)
Within group comparison				
*p*-value^*^	0.0566	0.0019	0.0473	0.0002
Mean change	0.30	0.74	0.29	0.91
95% CI	[− 0.01–0.61]	[0.29–1.19]	[0.00–0.57]	[0.45–1.36]
Between group comparison				
Mean change	−0.44		− 0.62	
95% CI	[−0.97–0.09]		[−1.14–0.10]	
*p*-value^**^	0.1057		0.0209	

*n* number of subject at each visit; *N* total number of subjects, *ITT* Intent to treat, *PP* Per protocol, ***Hb*** **Hemoglobin**

^*^ *p*-value were obtained using Paired t Test for mean (two tailed, **α = 0.05**)

^**^p-value were obtained using Unpaired t Test for mean change (two tailed, **α = 0.05**)

Note: Patients taken where Hb < 10 at Screening

Originally Table 5 was published as:

**Table 5:** Time to initially attained target Hb level (10–12 g/dL) and proportion of patients attained target Hb level (10–12 g/dL) at EOC and EOM

Parameter	ITT Population (*N* = 126)		PP Population (*N* = 93)	
	Darbepoetin alfa (*n* = 63)	Erythropoietin alfa (*n* = 63)	Darbepoetin alfa (*n* = 47)	Erythropoietin alfa (*n* = 46)
Number of weeks to initially attain target Hb				
Median (95%CI)	9.00 (7.00–11.00)	7.00 (4.00–9.00)	9.00 (7.00–10.00)	7.00 (4.00–8.00)
No. of Patients initially attained target Hb level				
N (%)	44 (78.57)	43 (82.69)	40 (85.10)	41 (89.13)
Hazard Ratio (95%CI)	0.807 (0.53–1.23)		0.778 (0.50–1.21)	
*P* Value	0.3212		0.2608	
No. of patients attained target Hb level at EOC				
N (%)	33 (52.38)	31 (49.2)	32 (68.08)	32 (69.56)
Odd ratios (95%CI)	0.9559 (0.46–1.99)		0.9410 (0.39–2.30)	
*P* value	0.9038		0.8938	
No. of patients maintained target Hb level at EOM				
(%)	24 (38.10)	36 (57.14)	15 (34.09)	23 (57.50)
Odd ratios (95%CI)	0.5748 (0.26–1.25)		0.4567 (0.17–1.22)	
*P* Value	0.1621		0.1180	

*EOC* End of correction, *EOM* End of maintenance, *ITT* Intent to treat, *PP* Per protocol, *Hb* Hemoglobin

The correct version of Table 5, with the corrected sections indicated in bold:

**Table 5:** Time to initially attained target Hb level (10–12 g/dL) and proportion of patients attained target Hb level (10–12 g/dL) at EOC and EOM

Parameter	ITT Population (*N* = 126)		PP Population (*N* = 93)	
	Darbepoetin alfa (*n* = 63)	Erythropoietin alfa (*n* = 63)	Darbepoetin alfa (*n* = 47)	Erythropoietin alfa (*n* = 46)
Number of weeks to initially attain target Hb				
Median (95%CI)	9.00 (7.00–11.00)	7.00 (4.00–9.00)	9.00 (7.00–10.00)	7.00 (4.00–8.00)
No. of Patients initially attained target Hb level				
N (%)	44 (78.57)	43 (82.69)	40 (85.10)	41 (89.13)
Hazard Ratio (95%CI)	0.807 (0.53–1.23)		0.778 (0.50–1.21)	
***p*-value**	0.3212		0.2608	
No. of patients attained target Hb level at EOC				
N (%)	33 (52.38)	31 (49.2)	32 (68.08)	32 (69.56)
Odd ratios (95%CI)	0.9559 (0.46–1.99)		0.9410 (0.39–2.30)	
***p*-value**	0.9038		0.8938	
No. of patients maintained target Hb level at EOM				
(%)	24 (38.10)	36 (57.14)	15 (34.09)	23 (57.50)
Odd ratios (95%CI)	0.5748 (0.26–1.25)		0.4567 (0.17–1.22)	
***p*-value**	0.1621		0.1180	

*EOC* End of correction, *EOM* End of maintenance, *ITT* Intent to treat, *PP* Per protocol, *Hb* Hemoglobin

Originally the last paragraph of the ‘Safety assessment’ section was published as:

- Altogether, DA-α had a similar safety profile to that of EPO and no antibody formation was identified.

The correct presentation of the last paragraph of the ‘Safety assessment’ section, with the corrected words indicated in bold:

- Altogether, DA-α had a similar safety profile to that of EPO and no **anti-drug antibody** formation was identified.

Originally two sentences in the first paragraph of the ‘Discussion’ section were published as:

- Evaluating the iron availability for erythropoeisis is crucial in treating anaemia patients with CKD.Iron deficiency can interfere with the response to EPO and DA-α and affecting the efficacy

The correct presentation of two sentences in the first paragraph of the ‘Discussion’ section, with the corrected words indicated in bold:

- Evaluating the iron availability for erythropoeisis is crucial in treating anaemia patients with **CKD. Iron** deficiency can interfere with the response to EPO and DA-α and affecting the efficacy
