# Supplementary material for: Efficacy, tolerability and safety of darbepoetin alfa injection for the treatment of anemia associated with chronic kidney disease (CKD) undergoing dialysis: a randomized, phase-III trial
Source: BMC Nephrol. 2019 Mar 13;20:90. doi: 10.1186/s12882-019-1209-1 (PMC6417108; doi:10.1186/s12882-019-1209-1)
Supplement: Supplementary file 1 — Table S1. List of Institutional Ethics Committee. (DOCX 14 kb) [file 12882_2019_1209_MOESM1_ESM.docx]

**Additional file 1: Table S1. List of Institutional Ethics Committee**

| Name and Address of Ethics Committee | Approval Date |
| --- | --- |
| Ethics committee, Apollo Hospital, Jubli Hills, Hyderabad | 15-Jun-12 |
| Institutional Ethics Committee, CARE Foundation, CARE Hospital, Road No.01, Banjara Hills, Hyderabad – 500 034 | 12-Jan-12 |
| Medictiti Ethics Committee, Mediciti Hospitals, Mediciti Institute of Medical Sciences & Share Organization, Mediciti Hospitals, 5-9-22, Near Sarovar Hotel, Secretariat Road, Hyderabad - 500063 | 2-Feb-12 |
| Maarg Independent Ethics Committee,  Plot No. 38, P&T colony, Near RTA Office, Trimulgherry, Secunderabad - 15 | 17-May-12 |
| Tanker Foundation Ethics Committee, 17, Wheatcroft Road, Nungambakkam, Chennai-600034 | 6-Jun-12 |
| Institutional Ethics Committee, Ashwini Critical and Heart Care Centre Pvt. Ltd, Nanded | 7-Nov-12 |
| Institutional Ethics Committee, B.Y.L Nair Ch.Hospital & T.N. Medical College, G-Building, Ground Floor, Dr. A.L. Nair Road,  Mumbai- 400008, Maharashtra | 10-Sep-12 |
| Astha Independent Ethics Committee, Ahemdabad, Gujarat. | 18-Feb-12 |
| Muljibhai Patel Society For Research in Nephro-urology Ethics Committee, Nadiad, Gujarat | 9-Jan-12 |
| Clinical Research Ethics Committee, Medica Superspecialty Hospital, 127, Mukundapur, E.M. Bypass, Kolkata-700099, West Bengal | 20-Dec-11 |
| Institutional Ethics Committee, Institute of Post Graduate Medical Education & Research, 244, AJC Bose Road, Kolkata-700020, West Bengal | 13-Aug-12 |
| Adarsh Independent Ethics Committee, Ahemdabad, Gujarat | 18-Jan-13 |
| Swapn Ethics Committee, Ahemdabad, Gujarat | 12-Jan-13 |
| ACE Hospital and Research Centre, Pune, Maharashtra | 22-Jan-13 |
